# Evaluating the low-rank coal degradation efficiency bioaugmented with activated sludge

**DOI:** 10.1038/s41598-024-64275-2

**Published:** 2024-06-27

**Authors:** Marzhan Kozhakhmetova, Nuraly Akimbekov, Ilya Digel, Kuanysh Tastambek

**Affiliations:** 1 Al-Farabi Kazakh National University, 050040 Almaty, Kazakhstan; 2 Khoja Akhmet Yassawi International Kazakh-Turkish University, 161200 Turkestan, Kazakhstan; 3 West Kazakhstan Marat Ospanov Medical University, Maresyev Str. 68, 030019 Aktobe, Kazakhstan; 4 Aachen University of Applied Sciences, Heinrich-Mussmann-Straße 1, 52428 Jülich, Germany; 5 M. Auezov South Kazakhstan University, 160012 Shymkent, Kazakhstan

**Keywords:** Low-rank coal, Activated sludge, Microbial community, Biodegradation, Humic-like compounds, Biotechnology, Microbiology, Ecology, Environmental sciences

## Abstract

Microbial bioaugmentation of coal is considered as a viable and ecologically sustainable approach for the utilization of low-rank coals (LRC). The search for novel techniques to derive high-value products from LRC is currently of great importance. In response to this demand, endeavors have been undertaken to develop microbially based coal solubilization and degradation techniques. The impact of supplementing activated sludge (AS) as a microbial augmentation to enhance LRC biodegradation was investigated in this study. The LRC and their biodegradation products were characterized using the following methods: excitation-emission Matrices detected fluorophores at specific wavelength positions (O, E, and K peaks), revealing the presence of organic complexes with humic properties. FTIR indicated the increased amount of carboxyl groups in the bioaugmented coals, likely due to aerobic oxidation of peripheral non-aromatic structural components of coal. The bacterial communities of LRC samples are primarily composed of *Actinobacteria* (up to 36.2%) and *Proteobacteria* (up to 25.8%), whereas the *Firmicutes* (63.04%) was the most abundant phylum for AS. The community-level physiological profile analysis showed that the microbial community AS had high metabolic activity of compared to those of coal. Overall, the results demonstrated successful stimulation of LRC transformation through supplementation of exogenous microflora in the form of AS.

## Introduction

Coal is among the most significant sources of energy on a global scale. As daily depletions of high-rank coal occur worldwide, the utilization of lower-grade raw materials for fuel purposes becomes increasingly vital. However, the high levels of ash and moisture in low-rank coal (LRC) pose serious environmental issues^1^. According to the International Codification System for low-rank coal utilization, LRC includes lignite (C + B type coals) and sub-bituminous coal (A type coals). This category of coals is defined as having a vitrinite mean random reflectance percent in oil (Ro%) less than 0.6% and a gross calorific value (GCV) computed on a "moist, ash-free" basis (m, af) less than 24 MJ/kg^2^.

Modern coal processing technologies are increasingly focused on generating value-added products and while mitigating environmental impact. Controlled coal degradation enables the production of a diverse range of beneficial products, including desulfurized coal, humic substances and organic fertilizers. This can be achieved through the microbial degradation^3,4^. Microbial technologies exhibit superior performance compared to physical and chemical coal processing due to their minimal technical requirements and environmental friendliness^5^. However, the remarkably heterogeneous composition of coal presents challenges to this approach^6^. Numerous published studies are in accord that, despite the great potential of coal biosolubilization, its exact mechanisms often remain unknown owing to the diversity of the involved microorganisms^7^. The pioneering work of Facuss in 1981 demonstrated the potential of using coal as a substrate for bacteria, followed by Cohen and Gabriele's identification of fungi capable of dissolving highly oxidized lignite into liquid form^8,9^. These works laid the foundation for further studies of microbial degradation of coal^10–12^. In some cases, specific bacterial strains are employed to facilitate targeted coal transformations, such as desulfurization^13–15^. Ali et al. identified the genera *Bacillus* and *Kocuria* as promising candidates for coal degradation, based on their solubilization/decomposition performance and ligninolytic activity^16^. Furthermore, *Penicillium ortum* MJ51 was successfully employed to convert solid lignite to liquid value-added compounds. Within 8 days it solubilized 36.4% of raw lignite and 82.0% of nitric acid-treated lignite^17^.

Various pre-treatment techniques may be employed to enhance the susceptibility of LRC to microbial solubilization and to optimize the yield of liquid products. One of them is bioaugmentation, that uses pure microbial cultures or consortia from various sources to enrich native coal microbiota. It has been already observed in several studies that lignite pre-treatment based on bioaugmentation accelerates coal liquefaction^18–21^. Recent research highlights the effectiveness of bioaugmentation in enhancing the performance^22–24^ and interactions^25^ of microbial communities, enabling them to adapt more effectively to varying environmental conditions.

By developing and using novel microbial consortia through bioaugmentation, productivity and flexibility in producing specific product profiles can be further increased. Microbial consortia capable of liquefying or solubilizing coal have been identified in diverse environments, including coal mines, waste dumps, oil well settling ponds and filters, as well as freshwater and marine sediments^26^. One of fundamental aspects of liquefaction and biosolubilization lies in the production of secondary metabolites by microorganisms, particularly biosurfactants, which reduce the surface tension of coal and enhance its solubility^27^. Therefore, the impact of microbial surfactants on viscosity and emulsification characteristics of coal have been addressed in several studies^28,29^.

Our study primarily analyzes and contrasts the metabolic products before and after biodegradation of LRC bioaugmented with AS. By investigating how microorganisms can alter the complex structure of coal, this research aims to comprehensive understand of the role of bioaugmentating microorganisms in generating value-added products through coal conversion. The LRC samples from the Ekibastuz, Oi-Qaragai, and Karaganda coal basins in Kazakhstan were selected for this study due to their elemental and technical properties, which present interesting opportunities for microbial degradation. Specifically, significant amount of organic compounds in the samples may be beneficial for their better biotransformation^30,31^. Activated sludge was chosen as the source of exogenous augmenting microflora due to its robust metabolic potential^32^, abundant microbial community^33^ and ability to withstand environmental stress factors^34^.

It should be expected that after the addition of AS, the level of metabolic activity of the LRC microflora will significantly increase, which will contribute to a more efficient transformation of coal into humic acids, a decrease in aromaticity and a significant increase in the number of carboxyl and carbonyl functional groups.

## Materials and methods

### Research materials

#### Coal samples

LRC samples were collected according to ISO 18283:2006 “Hard coal and co-sampling” and ISO 13909-4:2016 “Preview Hard coal and co-mechanical sampling. Part 4: Coal – Preparation of test samples” from three different regions of Kazakhstan, including (1) Ekibastuz coal basin—EKI (51° 43′ 52.1″ N 75° 24′ 23.7″ E), Pavlodar province, (2) Oi-Qaragai coal basin—OIQ (43° 11′ 36.7″ N 80° 35′ 49.2″ E), Almaty province, and (3) Karaganda coal basin—KAR (49° 51′ 48.5″ N 73° 06′ 17.7″ E), Karaganda province. The raw coal was pulverized and sieved to a particle size of 150 µm thought a grinder prior to use.

#### Activated sludge samples

Activated sludge (AS) as the source of exogenous microflora, was obtained from the wastewater treatment system in Almaty, Kazakhstan (43.219088, 76.918925). Samples were collected according to ISO 5667-13:2011 “Water quality—Sampling—Part 13: Guidance on sampling of sludges”. The enrichment and pre-acclimatization methods as well as culture medium composition were performed as described in our previous work^35^.

### Research methods

#### Proximate and ultimate analysis

LRC samples were analyzed using standard analytical methods to determine their physical and chemical characteristics. The Vario EL III CHNS analyzer (Elementar GmbH, Germany) was used for the elemental (C, H, N, and S) analysis. The functional composition of combustion products of coal samples was measured using an S6 JAGUAR XRF wave X-ray spectrometer (Bruker, Germany). The spectrometer was equipped with an X-ray tube with a Rh anode, and a rated power of 4 kW. The H/C and O/C ratios are determined by dividing the weight percentage of each element by its atomic weight, and then dividing the resulting values for hydrogen and oxygen by the value for carbon^36^.

#### Determination of pH

Coal acidity was determined using a SevenExcellence™ pH meter (Mettler Toledo, Switzerland). Before measurements, the pH meter was calibrated using three different types of standard pH buffer solutions (pH values of 4.01, 7.00, and 9.02; Mettler Toledo). Prior to measurement, 20 g of the original carbon were dissolved in 50 ml of distilled water, exposed to ultrasonic treatment for an 1 h, allowed to equilibrate for six hours The solution was then filtered through a paper filter and examined^37^.

#### Microbial diversity analysis

##### DNA extraction and amplification

Genomic DNA was extracted in triplicate from all samples using AxyPrep™ Bacterial Genomic DNA (Axygen, Netherlands). The DNA integrity was checked by 1% agarose gel electrophoresis. Based on the designated sequencing region, specific primers 515F-806R (515F-GTGCCAGCMGCCGCGGTAA, 806R-GGACTACHVGGGTWTCTAAT) with barcodes are synthesized. DNA polymerase TransGen AP221-02 was used for PCR. The PCR amplification was carried out using a PCR instrument ABI GeneAmp® 9700. All samples were processed according to the established standard operation protocols, and each sample was amplified in triplicate. The PCR products were examined by electrophoresis in a 2% agarose gel. The AxyPrep DNA Gel Recovery Kit (AXYGEN) was used to cut the gel and extract the PCR products. Elution was carried out by Tris–HCl buffer (pH 7.0) and controlled by electrophoresis in 2% agarose.

#### Illumina sequencing

The Illumina MiSeq platform (Illumina, USA) was utilized to perform paired-end sequencing (2 × 300) on purified amplicons pooled in an equimolar ratio, in accordance with the standard methods defined by Majorbio Bio-Pharm Technology Co. Ltd. (China). Operational taxonomic units (OTUs) were grouped using UPARSE (version 7.1 http://drive5.com/uparse/) with a similarity criterion of 97%. The UCHIME algorithm was utilized to detect and eliminate chimeric sequences (https://drive5.com/uchime). Utilizing the RDP classifier algorithm (http://rdp.cme.msu.edu/) with a 70% confidence level, the taxonomy of every 16S rRNA gene sequence in the Silva 16S rRNA database (SSU123) was examined. The Majorbio I-Sanger Cloud Platform, a free web tool, was used for all bacterial community analyses (www.isanger.com).

#### Microbial treatment and microcosm construction

An individual microcosm was built in 250 ml Duran bottles for each type of coal. 25 g of coal from each sample and 5 g of pre-acclimatized AS was added to the E8 synthetic medium (0.7 g KH_2_PO_4_, 1.5 g (NH_4_)_2_HPO_4_, 0.8 g MgSO_4_, 0.5 g NaCl, and 1 L of distilled water). Preliminary aerobic incubation was carried out at 30 °C for 30 days. After fermentation, the supernatant was filtered with 0.22 μm membrane filter, and the sediment was subjected for further anaerobic cultivation under nitrogen. A modified medium (0.35 g L^−1^ KH_2_PO_4_, 0.23 K_2_HPO_4_ g L^−1^, 0.50 NH_4_Cl g L^−1^, 0.41 MgCl_2_ × 6H_2_O g L^−1^, 0.25 CaCl_2_ × 2H_2_O g L^−1^, 2.25 NaCl g L^−1^, 1.42 FeCl_2_ × 4 H_2_O mg L^−1^, 0.85 NaHCO_3_ g L^−1^, 0.30 C_3_H_8_ClNO_2_S × H_2_O g L^−1^, trace element solution SL-10 ml L^−1^, Wolin’s vitamin solution-10 ml.L^−1^) was used for this purpose as described in Ref.^38^. All components (except NaHCO_3_, C_3_H_8_ClNO_2_S, vitamins, and trace elements) were dissolved in 1 L distilled water. The mixture was bubbled with nitrogen for 1 h, tightly sealed, and sterilized by autoclaving at 121 °C and 1.2 bar for 20 min. NaHCO_3_, C_3_H_8_ClNO_2_S, vitamins, and trace elements were sterilized by filtration into oxygen-free sterile 250 mL Duran bottles^38^. Anaerobic cultivation was performed in an incubator shaker (EC-20/60, Biosan, Latvia) 50 rpm, 35 °C for 30 days.

#### Community level physiological profile (CLPP)

The metabolic functional diversity of the microbial communities in LRC and activated sludge samples was assessed using Biolog™ EcoPlate according to manufacturer instructions. The enzymatic activity and destructive potential of bacterial consortia that provide mutual support during coal decomposition have been the main subject of this methodological section. Thirteen distinct carbon sources were applied in triplicate on 96-well plates. For all carbon sources, the average well color development (AWCD) was calculated using the following formula^39^:

$${\text{AWCD}} = \sum\limits_{i = 1}^{n} {\frac{{C_{i} - C_{0} }}{n}}$$

n is the number of substrates, which is n = 31.

10 g coal samples and 1 g of AS were suspended in sterile peptone solution, agitated at 20 °C for 20 min, and then incubated at 4 °C for 30 min^40^. Further, the cultures were diluted to an optical density of 0.08 at 590 nm. 150 μl of supernatant was applied to a Biolog EcoPlate and then incubated at 25 °C^41^. Carbon substrate utilization was determined by measuring absorbance at 590 nm every 24 h for 96 h using a microplate reader (Bio-Rad 680, USA). The optimal range of optical density (OD) values is represented by the values attained after 96 h of incubation. Consequently, as a function of incubation time, statistical analysis of Shannon evenness (*E*) and Shannon richness (*R*) values were computed.

#### Fourier transform infrared spectroscopy (FTIR)

FTIR spectrometer ALPHA II QuickSnap (Bruker Optics GmbH, Germany) was used to record the spectra of raw and treated samples. Every filtered and dried coal sample was placed in the reaction chamber. From the reaction chamber's base, dry air entered and left at the top. A 4 cm^−1^ resolution scan was conducted over the range of 400–4000 cm^−1^. A set of spectra was obtained for every sample at intervals of 24 spectra per sec.

#### Fluorescence spectroscopy

JASCO FP-8500 (JASCO, Japan) spectrofluorometer was employed to detect fluorescent dissolved organic matters in the samples. The data fluorescence spectra were modeled with an excitation wavelength ranging from 200 to 400 nm (step 5 nm) and emission wavelength from 280 to 550 nm (step 10 nm). After incubation, all samples were centrifuged at 9000 g for 10 min and filtered through a membrane filter (0.45 μm). The supernatant was then stored at 4 °C for further analysis. The ratio of the emission fluorescence intensities at 470 and 520 nm for the excitation wavelength of 370 nm was used to compute the fluorescence index (FI). The biological index (BIX) was calculated by dividing the fluorescence intensity at excitation wavelength 310 nm and emission wavelength 380 nm by the fluorescence intensity emitted at 430 nm at excitation wavelength 310 nm.

#### Scanning *electron* microscope

Scanning electron microscopy was performed using Hitachi S-4800 SEM System (Japan) with a resolution of 1.0 µm for morphological characterization of coal samples. To prepare the samples, the powdered coal was first scattered into a metallic holder, and then sputter coated with gold. Initial configurations: current 30 mA and accelerating voltage 5 kV.

#### Statistical analysis

Statistical differences between groups were analyzed using one-way analysis of variance (ANOVA). The significance of differences between means was assessed using Fisher's least significant difference (LSD) with a significance level of 0.05. Unless otherwise stated, graphically presented values are means ± standard deviation (SD).

## Results and discussion

### Characterization of coal

Table 1 presents the results of the proximate and ultimate measurements conducted on coal samples from various regions. The moisture and ash contents of all coal samples were 9.7–11.8 (wt.%) and 12.2–37.9 (wt.%), respectively. The samples contained a moderate amount of volatile matter and fixed carbon. Sulfur concentrations in coals were negligible, falling short of one percent in all samples. These coals are classified as lignite-B in rank according to the H/C vs. O/C ratios^36,42^, having a heating value of 13.8–21.2 MJ/kg.

**Table 1:** Proximate and ultimate analysis and pH of coal samples.

Characteristics	Coal samples		
	EKI	OIQ	KAR
Moisture, % (W)	9.7	11.8	10.5
Ash, % (A)	37.9	12.2	23.1
Volatile matter, % (V)	27.1	35.8	41.7
Heat of combustion, MJ/kg (Q)	13.8	15.5	21.2
C	53.1	55.1	54.6
H	3.23	3.9	3.3
N	0.2	1.4	1.5
S	0.8	0.4	0.9
O	15.3	18.4	19.8
H/C	0.7	0.7	0.7
O/C	0.2	0.3	0.3
pH	7.37	6.84	7.94

The ash of coal samples was analyzed for the presence of principal components and microelements via the X-ray fluorescence, as detailed in Table 2. Oxides of silicon and aluminum constitute the largest proportion, so for EKI—58.61% and 21.79%, OIQ—36.11% and 19.20%, KAR—27.74% and 14.26%, respectively.

**Table 2:** Chemical composition of ash from the studied coal samples.

Components	Designation	Coal samples		
		EKI	OIQ	KAR
SiO_2_	%	58.61	36.11	27.74
Al_2_O_3_	%	21.79	19.20	14.26
Fe_2_O_3_	%	8.16	8.01	9.22
CaO	%	3.47	5.78	7.75
MgO	%	0.39	1.78	2.34
K_2_O	%	0.56	0.29	1.80
Na_2_O	%	0.11	1.28	2.84
P	ppm	26.40	39.44	48.15
Ti	%	0.02	0.01	< 0.01
Mn	ppm	15.31	68.01	90.00

### Characterization of coal microbial community

#### Microbial community structure

**Figure 1:**
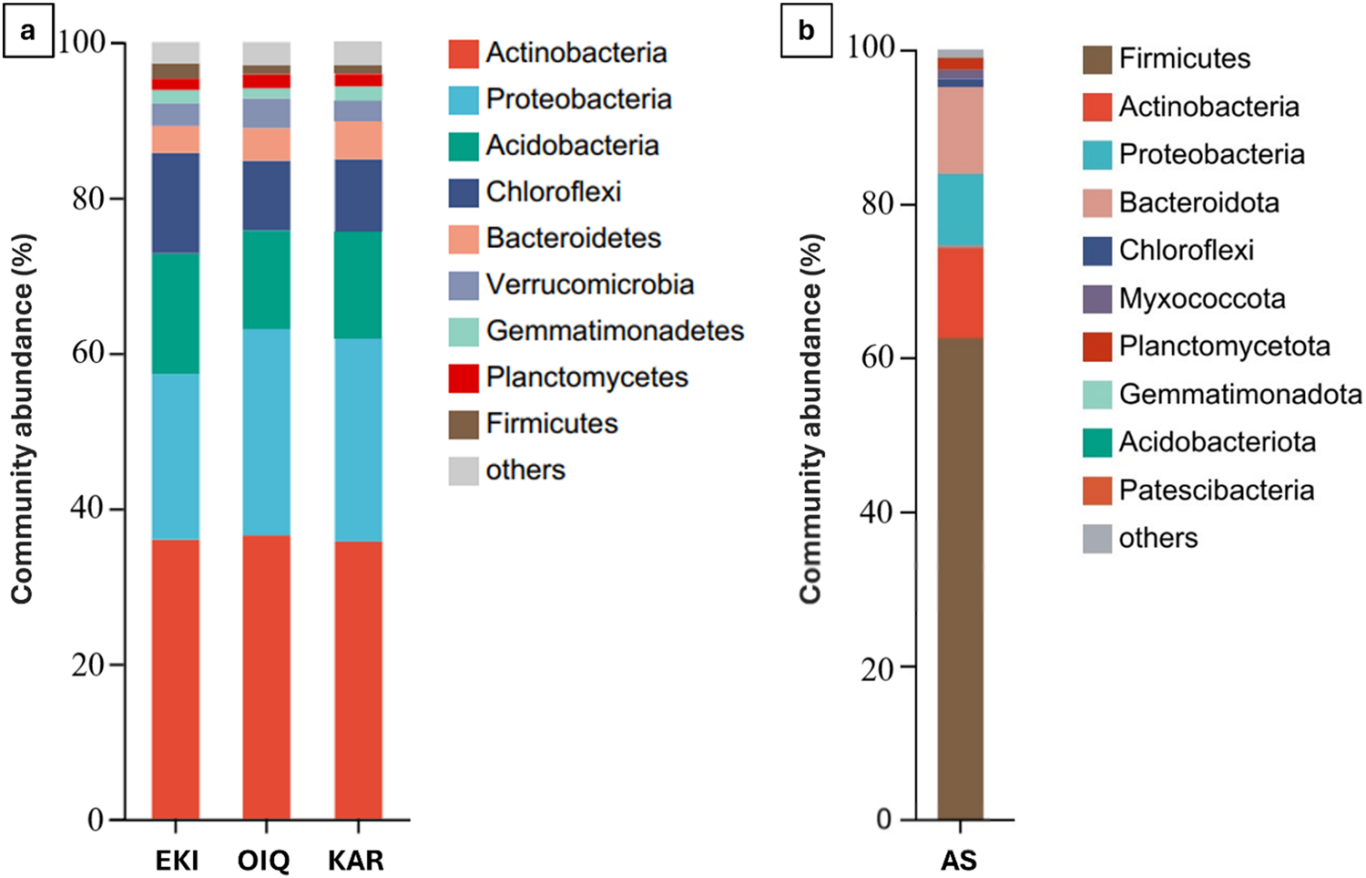
Bar-chart of the microbial relative abundance of coal samples (**a**) and AS (**b**) sample at phylum level.

The bacterial community composition analysis revealed the presence of eight major bacterial phyla (Fig. 1a). They consisted predominantly of the phyla *Actinobacteria*, and *Proteobacteria*. In all samples, sequences from the phylum *Actinobacteria* were dominant, followed by those from the phyla *Proteobacteria, Acidobacteria, Chloroflexi, Bacteroidetes, Verrumicrobia, Gemmatimonadetes.* Bacterial sequences from KAR belonged to the phyla *Actinobacteria* (36.2%), *Proteobacteria* (25.8%), and *Acidobacteria* (13.11%). Other notable phyla included *Chloroflexi* (10.78%), *Bacteroidetes* (5.87%), *Verrucomicrobia* (3.77%), *Gemmatimonadetes* (2.25%), *Planctomycetes* (2.13%) and *Firmicutes* (1.14%). In the other two samples, *Actinobacteria* was the dominant phylum, with a relative abundance of 36.07% in EKI and 37.14% in OIQ. The second most dominant phylum was *Proteobacteria*, whose relative abundance was 26.1% and 21.5% for OIQ and EKI, respectively. Other major phyla were distributed as follows: *Acidobacteria* (12.55% in OIQ, 14.58% in EKI), *Chloroflexi* (9.61% in OIQ, 13.52% in EKI), *Bacteroidetes* (5.29% in OIQ, 4.66% in EKI), *Verrucomicrobia* (4.51% in OIQ, 4.11% in EKI), and *Gemmatimonadetes* (2.1% in OIQ, 2.3% in EKI), *Planctomycetes* (2.65% in OIQ, 2.15% in EKI), and *Firmicutes* (1.12% in OIQ, 2.31% in EKI). There were no significant differences in the composition of the microbial composition of the three types of coals.

The most prevalent six phyla for AS at the phylum level were *Firmicutes* (63.04%), *Actinobacteria* (11.69%), *Proteobacteria* (9.91%), *Bacteroidota* (10.38%), *Chloroflexi* (1.49%), and *Planctomycetota* (1.54%) (Fig. 1b).

**Figure 2:**
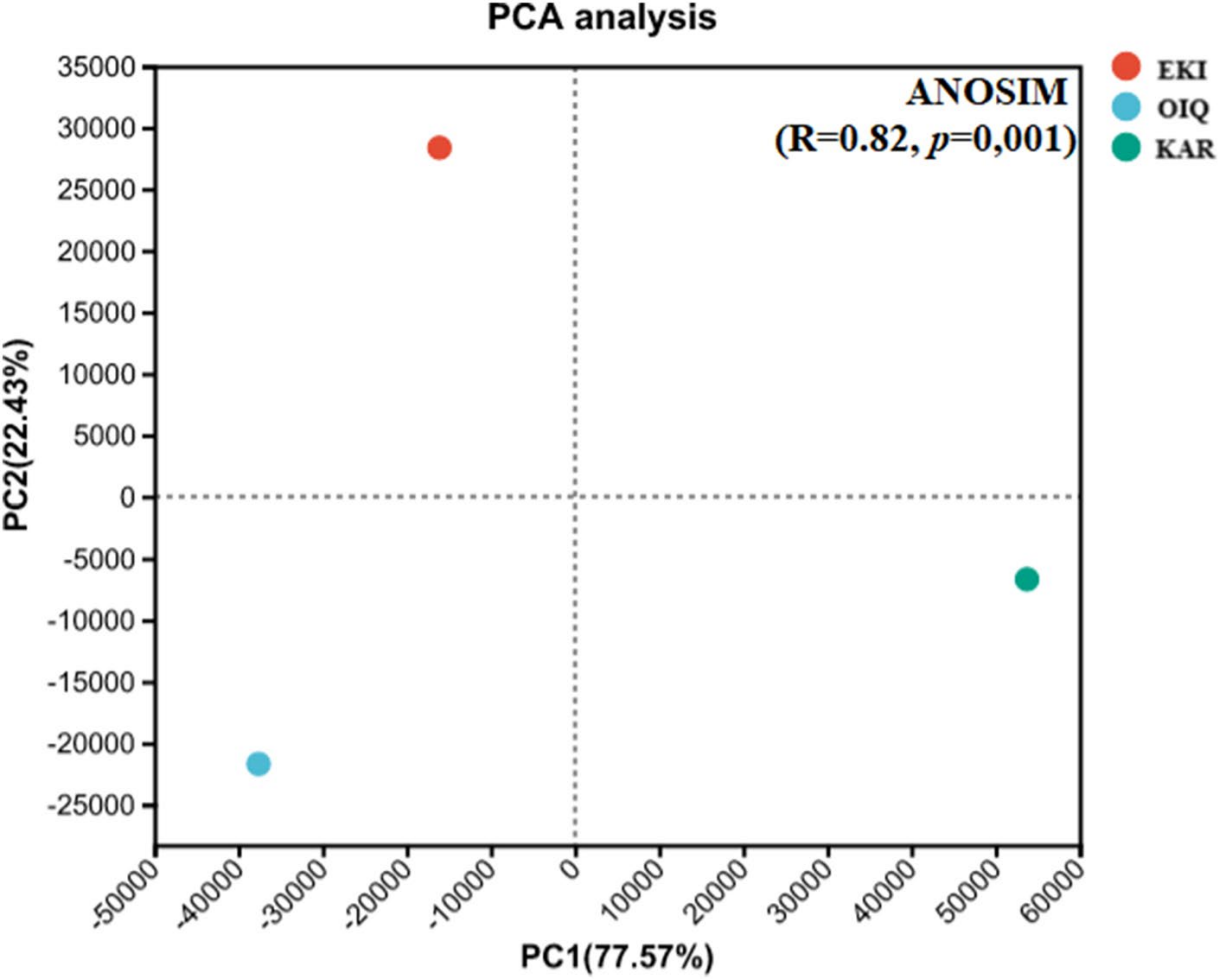
PCA analysis of the coal samples taxonomic relationships.

A principal component analysis was performed to observe the taxonomic relatedness at genus level among the three coal microbial communities. The more similar the community composition of the given coal samples, the closer they appear in the PCA plot (Fig. 2). Principal component 1 made a variance contribution of 22.43%, while principal component 2 contributed to 77.57%. After dimensionality reduction, differences in the metabolic characteristics of microbes in different coal samples were directly reflected in the location of points in space and could objectively and accurately explain the microbial diversity at the genus level. The coordinate comparison-based PCA results indicated that the three location points representing distinct coals were well separated in terms of relative distance.

**Figure 3:**
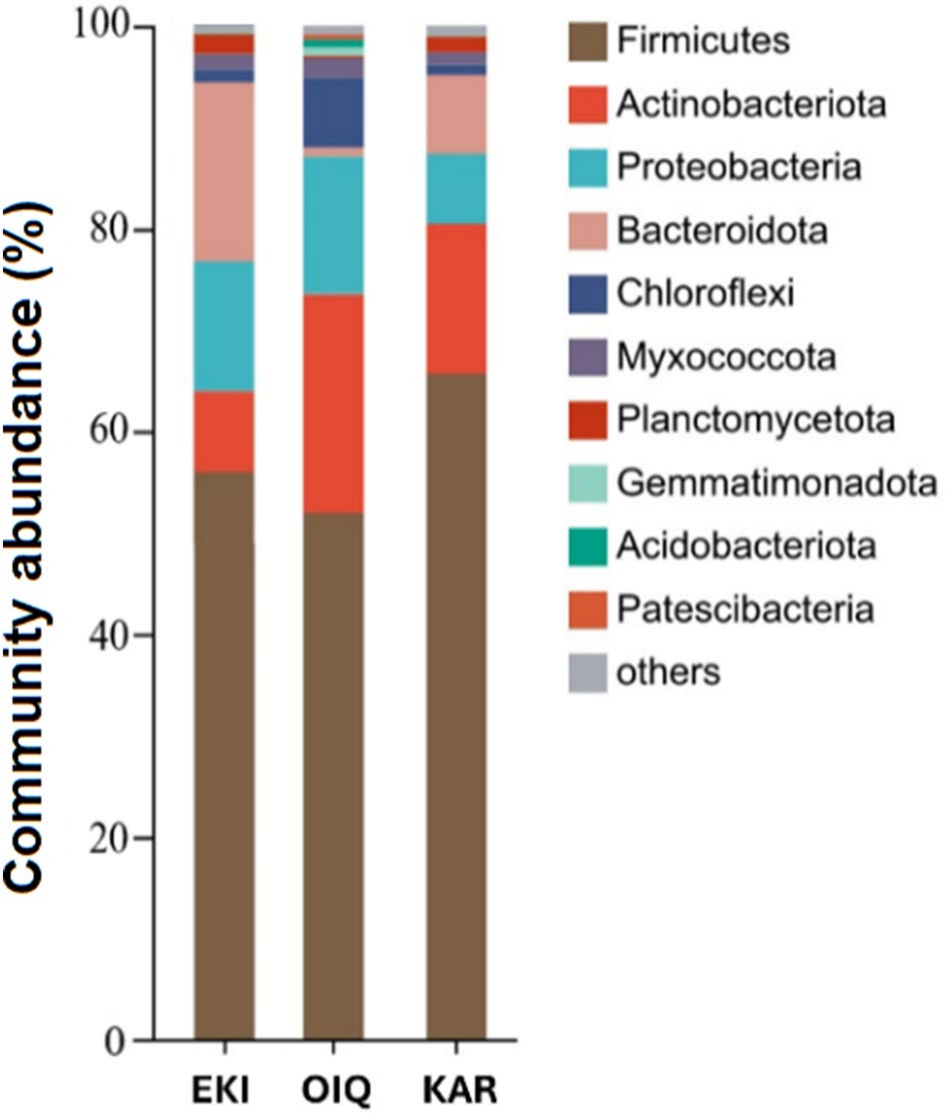
Bar-chart of the microbial relative abundance of bioaugmented coal samples at phylum level.

As visible in Fig. 3, bioaugmentation with AS dramatically changed the microbial community of coal samples compared to the raw coal samples. The phylum *Firmicutes* exhibited the most significant increments across all samples, as evidenced by increase of its relative abundance from 1.14 to 65.3%in EKI, from 1.12 to 51.4% in KAR, and from 2.31 to 55.1% in OIQ. *Proteobacteria* (7.62% in KAR and 19.25% in OIQ) and *Actinobacteria* (19.73% in KAR and 21.31% in OIQ, respectively) showed a decline in relative abundance after bioaugmentation and emerged as the secondary dominant phyla. The *Chloroflexi* and *Acidobacteria* in the KAR were significantly reduced after treatment, showing the dynamics from 13.11 to 1.31% and from 10.78 to 0.13%, respectively. Community abundance of the *Bacteroidota* (5.87% vs. 7.81%) in KAR and Chloroflexi (9.61% vs. 7.55%) in OIQ did not show significant differences before and after bioaugmentation. The relative abundance of the *Actinobacteria* and *Proteobacteria* in EKI decreased from 36.07 to 8.73% and from 21.5 to 12.33%. In the EKI the *Bacteroidota* grew significantly and became the subordinate phylum with relative abundance 17.89%, while *Chloroflexi* and *Acidobacteria* decreased significantly, from 14.58 to 0.09% and from 9.61 to 1.23%, respectively.

#### Microbial community-level physiological profile (CLPP)

**Figure 4:**
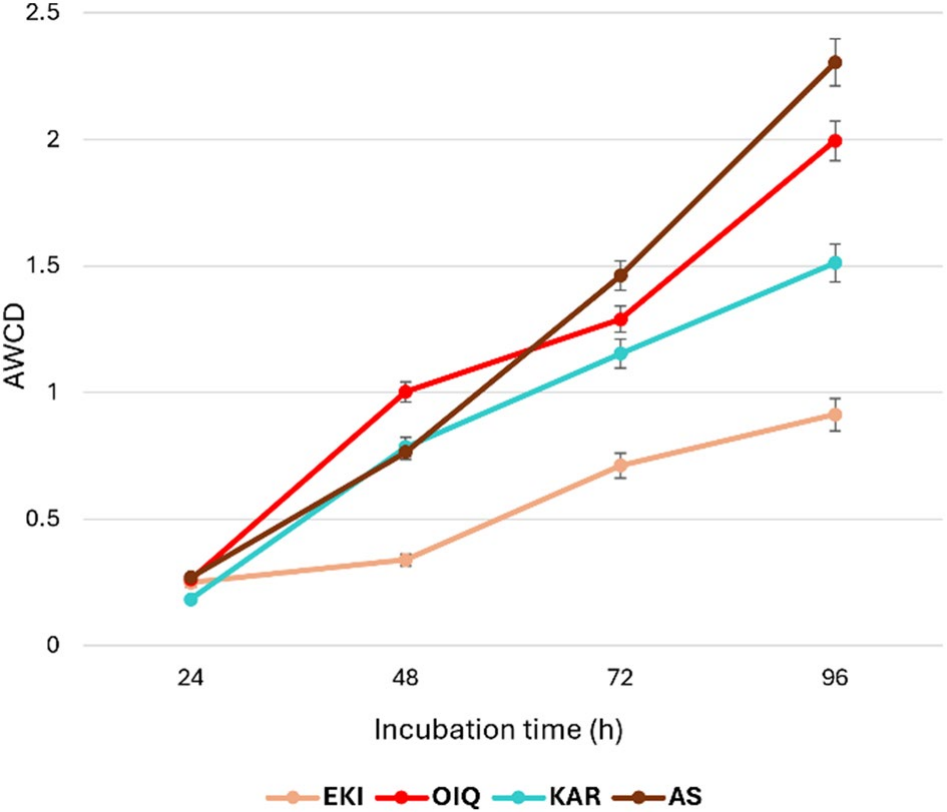
Community-level physiological profiles of coal and AS samples show as AWCD-dynamics.

The analysis of the CLPP data revealed that the bacterial community from coal samples utilized 31 carbon substrates. The average color development (AWCD) values for the AS and OIQ samples were 2.300 and 1.975, respectively, after 96 h. The EKI and KAR samples had AWCD values of 1.503 and 0.872, respectively. The EKI samples showed the lowest microbial activity and displayed AWCD dynamics with a distinct lag phase, followed by increase in AWCD values after 48 h (Fig. 4). The observed differences in AWCD curves may be attributed to differences in the inoculum density and community structure in the samples^43^.

**Figure 5:**
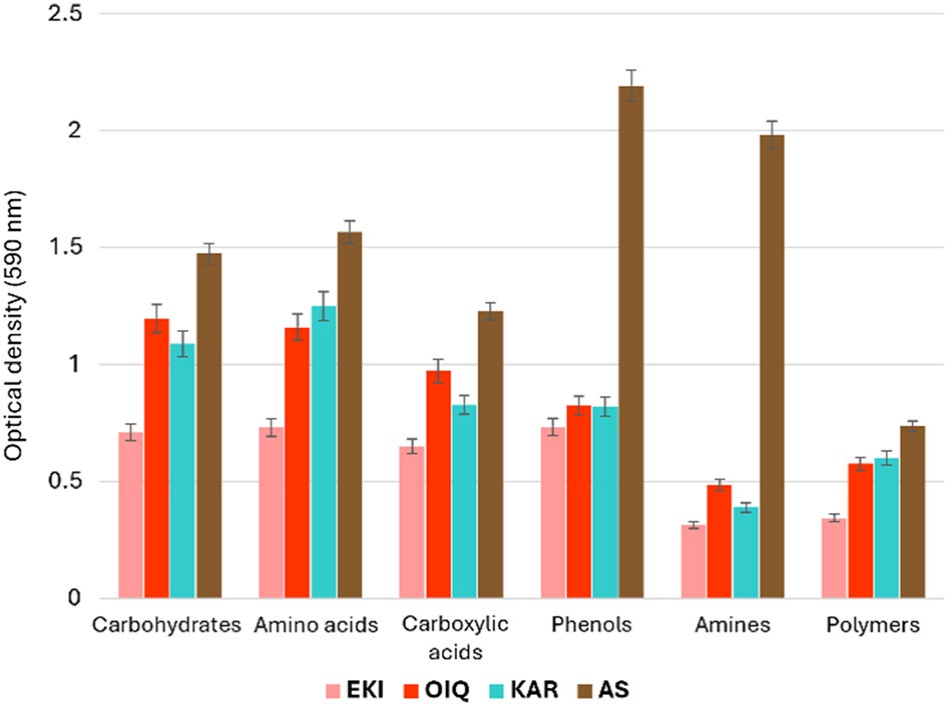
Community-level physiological profiles of coal and AS samples based on average carbon substrate utilization.

The utilization capability of six categories of substrates (carbohydrates, amino acids, carboxylic acids, polymers, amines, and phenols) which was highest in the activated sludge sample compared with coal samples, where the utilization rate was quite low, is shown in Fig. 5. The microbial communities in activated sludge were found to utilize phenols and amines more commonly than other substrates. While, the higher degree of carbohydrate and carboxylic acid utilization was more closely associated with the microbial communities of coals. The Shannon evenness (E) and richness (*R*) values obtained using the Biolog Ecoplate analysis are shown in Table 3.

**Table 3:** Shannon *E* and *R* indices based on substrate utilization patterns by initial microbial communities of the coal and AS samples.

Index	EKI	KAR	OIQ	AS
Shannon evenness (*E*)	0.995 ± 0.001	0.997 ± 0.001	0.997 ± 0.001	1.113 ± 0.001
Shannon richness (*R*)	26.97 ± 0.57	28.98 ± 0.51	28.98 ± 0.62	30.97 ± 0.59

Richness is defined as the total amount of carbon substrates used by microorganisms, and evenness is defined as the evenness of substrate use among all substrates used. Shannon evenness (E) did not vary between coal samples, with the exception of higher index level for AS sample. The greatest differences between samples were demonstrated by Shannon richness (R) measurements, where EKI coal sample have lowest richness value, while AS sample have highest level (Table 3). Higher richness indices imply higher oxidation levels of the carbon substrates.

**Figure 6:**
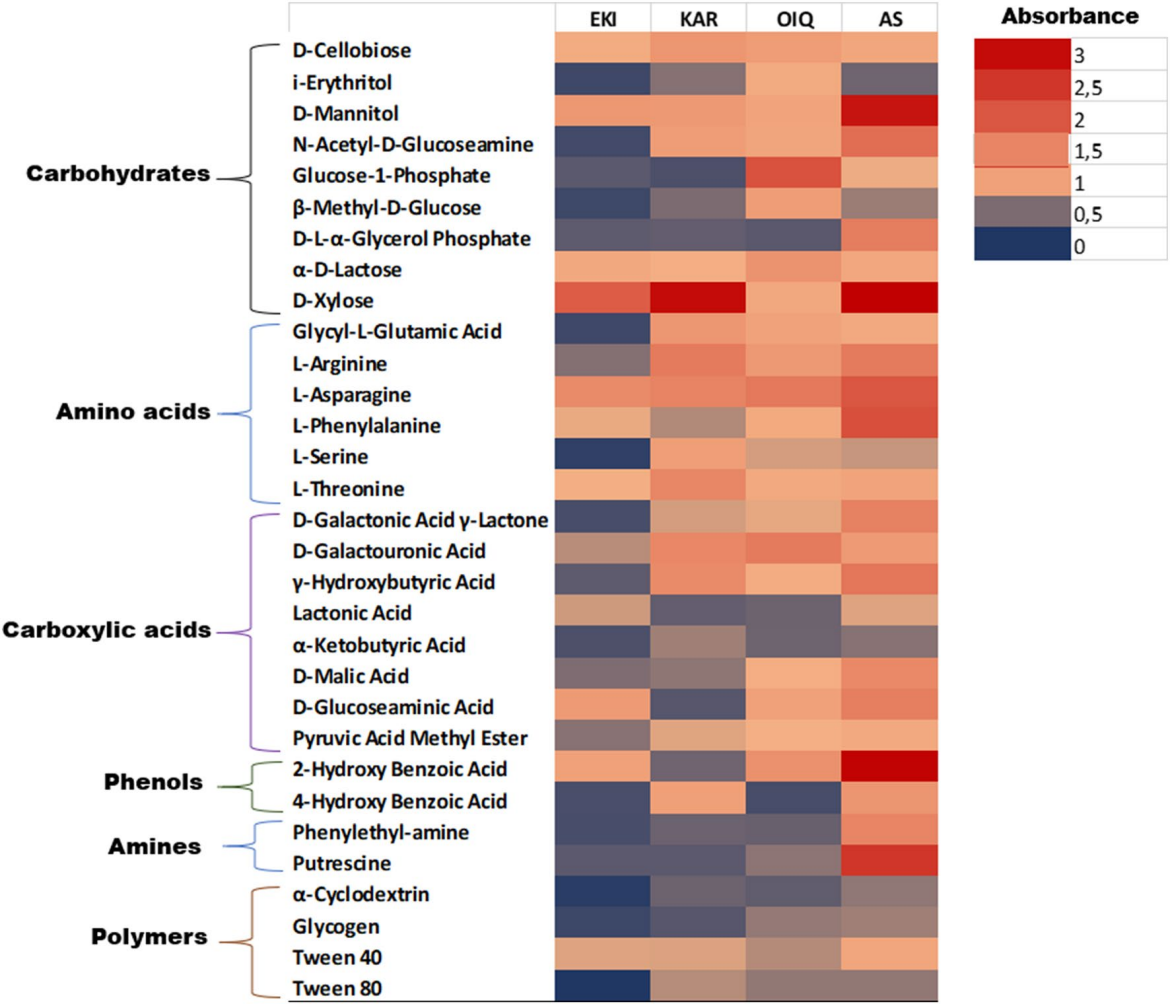
Heatmap created using data from 31 carbon sources examined in Biolog EcoPlates. The results illustrated the difference in microbial communities in each sample based on utilizing the same substrate. Based on the scale, red color denotes the maximum substrate utilization, while blue color represents the lowest utilization. The values on the scale (from 0.2 to 3) are the standardized OD values at 590 nm (p < 0.05).

In general, coal microbial communities were capable of utilizing each of the 31 carbon sources. A greater tendency for utilizing carbohydrates (d-lactose, β-methyl-d-glucoside, d-cellobiose, d-mannitol, d-xylose, i-erythritol, *N*-acetyl-d-glucosamine) and amino acids (l-arginine, l-asparagine, l-phenylalanine, l-serine, l-threonine, and glycyl-l-glutamic acid) was observed in coal microorganisms (Fig. 6). The highest microbial growth in AS was observed in phenolic substrates (2-hydroxy benzoic acid and 4-hydroxy benzoic acid).

According to the heatmap analysis, l-asparagine was the most actively utilized amino acid, whereas d-xylose was the most effectively utilized carbohydrate across all samples (Fig. 6). In general, the lowest utilization rates were typical for amines/amides (phenylethylamine and putrescine) and polymers. Remarkably, AS microbial community utilized more amines than those of coal samples^40^.

### Characterization of the bioaugmented coal products

#### Excitation-emission matrix fluorescence spectra

**Figure 7:**
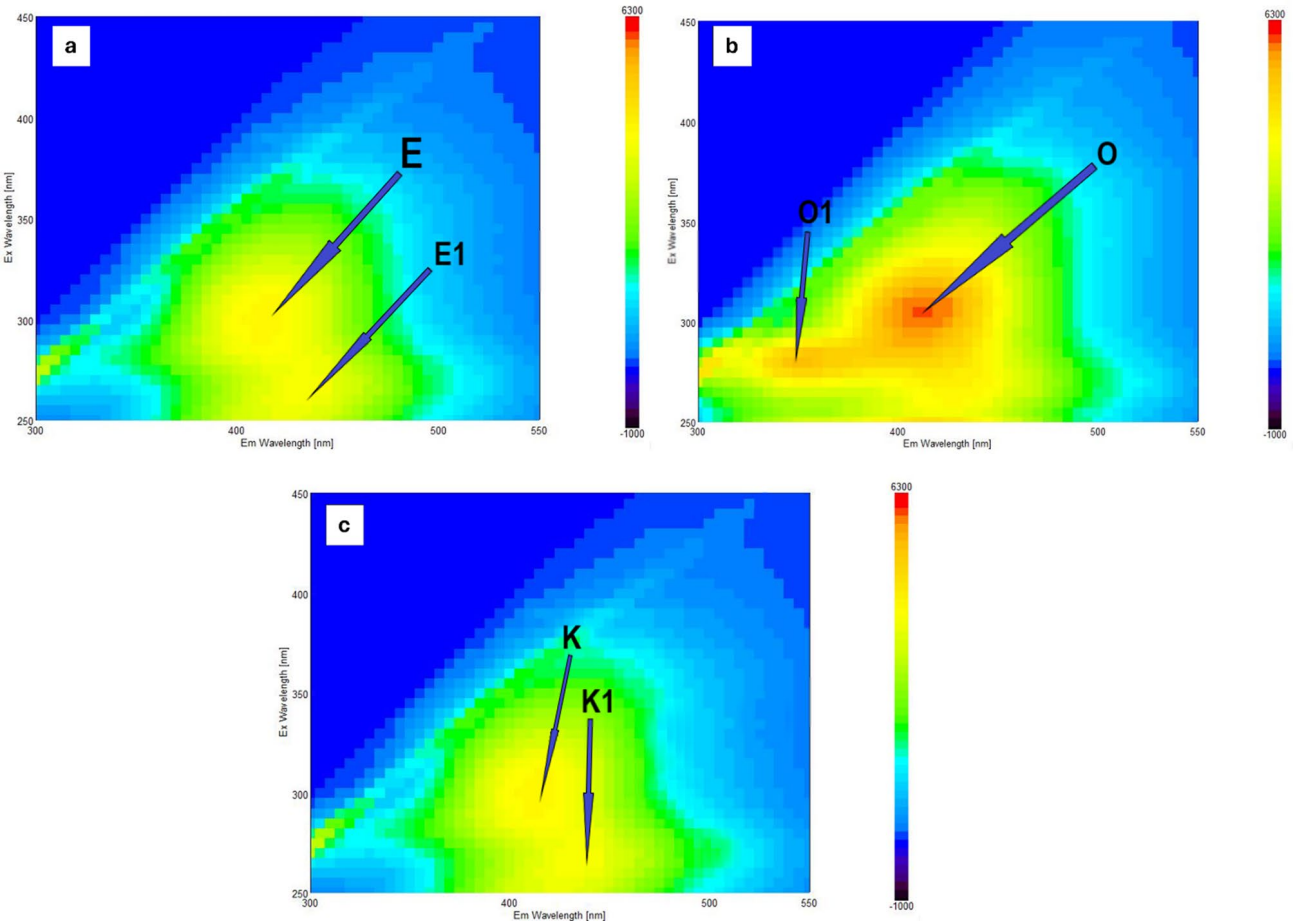
EEM spectra of the AS-bioaugmented of coal samples: (**a**) EKI, (**b**) OIQ and (**c**) KAR.

Matrix-based fluorescence excitation-emission spectroscopy of bioaugmented coal samples has detected humin-like fluorophore activity (Fig. 7). The OIQ exhibited the highest fluorescence intensity (maxima at 305 nm and 500 nm) at the excitation wavelength 250 nm. Such a EEM pattern is typical for humic or fulvic compounds^44^. The comparison of fluorescence spectra of KAR and EKI revealed just negligible dissimilarity between them.

Using the EEM model interpretation for dissolved humic organic matter, the fluorescence activities were evaluated at six specific wavelength coordinates denoted as peaks E, O, K, E1, O1, and K1 (Fig. 7) with maximum intensity at peak E (Ex/Em = 310/425 nm), peak O (Ex/Em = 310/415 nm), peak K (Ex/Em = 300/ 420 nm), peak E1 (Ex/Em = 255/440 nm), peak O1 (Ex/Em = 280/350 nm) and peak K1 (Ex/Em = 270/440 nm). Peaks O, E, and K indicate the presence of humic-like organic compounds^45^, whereas peaks O1, E1 and K1 might arise due to the presence of protein-like substances^46,47^.

The fluorescence indices (FI and BIX) were selected to examine the origins (terrestrial/microbial) of organic compounds and the influence from autochthonous biological activity^48^. The FI value of all samples was about 1.9, which correspond to microbial sources of organic matter with lower aromatic carbon content^49^. The BIX evaluates the contribution of autochthonous or biological (microbial) activity in the formation of organic compounds, mainly humic substances. Here, BIX values were within the range of 0.7–0.8, indicating the presence of biologically generated humic compounds^50^.

#### FTIR spectroscopic characterization

**Figure 8:**
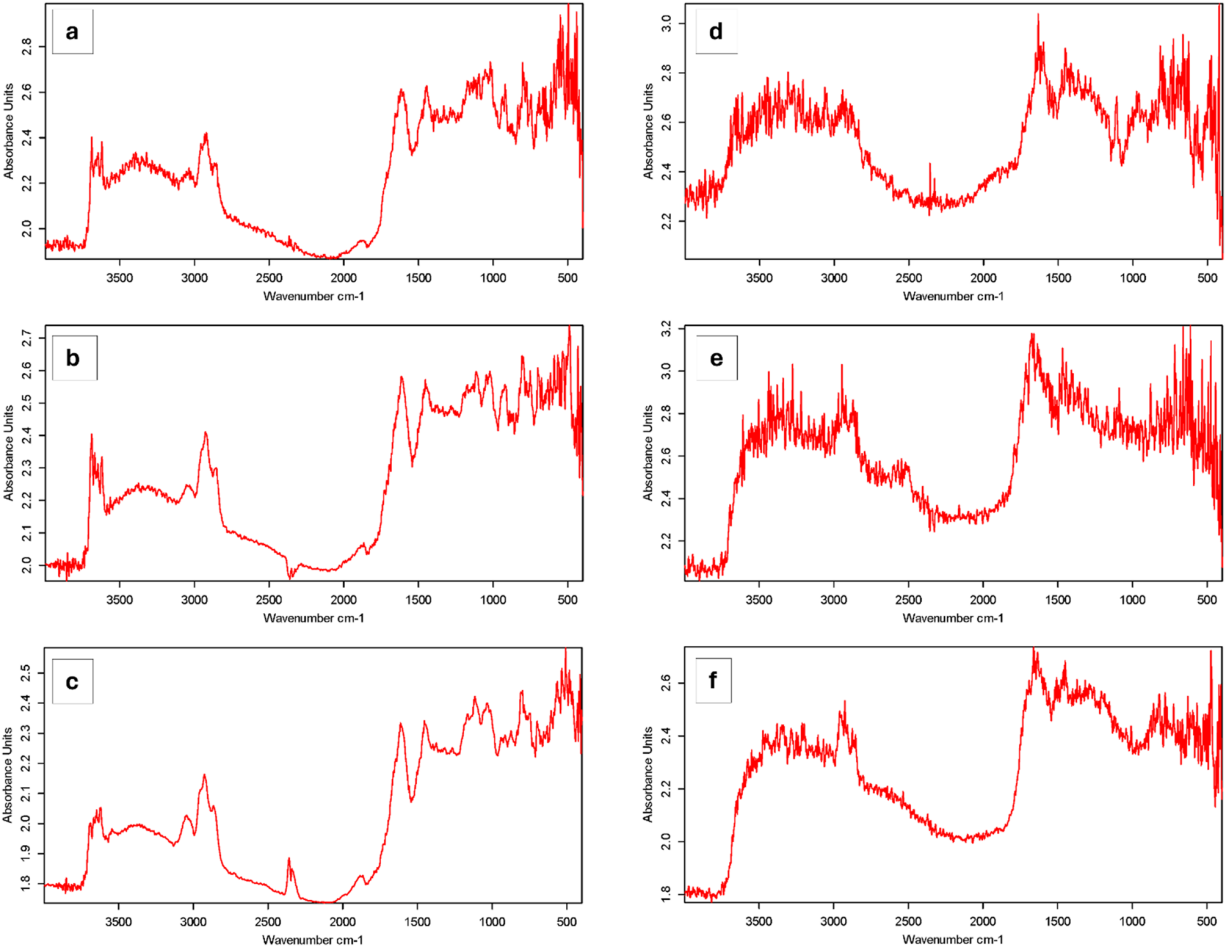
The FT-IR spectra of raw (**a** EKI, **b** OIQ, **c** KAR) and bioaugmented (**d** EKI, **e** OIQ, **f** KAR) coal samples.

The results of infrared spectroscopy of the indigenous coal samples are shown in Fig. 8a–c. The broad and powerful hydroxyl absorption (–OH, 3400–3200 cm^−1^) directly correlates with the moisture content of the coal^51^. The vibration of aliphatic hydrogen produced a prominent and acute absorption peak at 2926 cm^−1^. The band at around 1614 cm^−1^ may be attributed to stretching vibrations in the aromatic rings C=O and C=C. Around 1453 cm^−1^, a visible band is generated due to the symmetric aliphatic C–H vibration exhibited by the methylene (CH_2_) and methoxy (OCH_3_) groups^52^. Short peaks were found at 1114 and 1022 cm^−1^, seemingly corresponding to Si–O, C–O and C–O–R structures. The primary source of the absorption zone in the wavenumber range of 900–700 cm^−1^ is the bending vibrations of polysubstituted aromatic compounds^53^. The peaks seen within the 400–600 cm^−1^ range can be attributed to clay and silicate minerals^54^.

In general, four main functional groups can be distinguished in the primary coal samples, including hydroxyl, oxygen-containing, aliphatic, and aromatic structures. When the FTIR results of three distinct varieties of coal are compared, no substantial difference is seen except more pronounced aromaticity of the organic matter in the OIQ samples.

In the EKI samples bioaugmented with AS, multiple functional group modifications were observed (Fig. 8d–f). The IR spectra of the modified coals revealed the presence of visible alterations in the stretching vibrations of the OH group: in the free form it occurred within the range of 3619–3489 cm^−1^. A weaker intensity was observed at 3320 cm^−1^ and the vibrations of C–H bonds of aromatic compounds were seen at 3037 cm^−1^. In the region of 2853 and 2920 cm^−1^, vibrations of C–H bonds of alkanes became noticeable. An intense absorption band at 1615 cm^−1^, which can be attributed to vibrations of the C=C bonds in the aromatic ring or the C=O bond in carboxyls, signalized enrichment with oxygen-containing groups. Noticeable absorption around 1440 cm^−1^ indicated the presence of methyl groups. At the 1320 cm^−1^, weak absorption bands of vibrations of bonds with the C–O–H group were discernible. Planar deformation vibrations of C–H bonds of aromatic compounds were detected in the region of 1094 and 1019 cm^−1^. Absorption bands in the range 591–414 cm^−1^ can be possibly attributed to organometallic compounds.

The intense absorption in the region of 3500–3300 cm^−1^ of OIQ spectra after bioaugmentation was also altered; that may be attributed to the appearance of several bound- and free hydroxyl groups. The band at 1634 cm^−1^ denoted the most prominent =O and –COO groups. Absorption peaks in the region of 1548 cm^−1^ and 1408 cm^−1^ were also most noticeable, showing stretching vibrations of aromatic rings and asymmetric bending vibrations of –CH_2_ and –CH_3_ groups. In-plane C=H bending vibrations of aromatic compounds were detected at 1089 cm^−1^, 1043 cm^−1^, and 999 cm^−1^. Out-of-plane bending vibrations of aromatic compounds were observed in the region of 840–750 cm^−1^. The results indicate the formation of complex structures in the organic mass of coals, such as oxygen-containing carbonyl and ether functional groups.

The IR spectra of KAR show the absorption spectra at 3473–3211 cm^−1^ range, which corresponds to the stretching vibrations of –OH groups. In the 2925–2875 cm^−1^ range, there are strong spectral peaks originating from symmetrical stretching vibrations of methyl (–CH_3_) and methylene (=CH_2_) groups. The spectral features in the 1659–1634 cm^−1^ range correspond to the frequency of stretching vibrations of C=H bonds in alkenes, whereas 1449 cm^−1^ indicates stretching vibrations of methyl groups. The absorption spectra at 1315 cm^−1^ correspond to vibrations associated with the C–O–H group could be primary alcohol. Additionally, bending vibrations of the C–H bonds of the aromatic ring are visible at 857–772 cm^−1^. Vibrations of organometallic compounds occur within the range of 566–408 cm^−1^. Collectively, the IR-spectral measurements indicated dramatic increase in the diversity and concentration of functional groups after bioaugmentation, especially related to carbonyl, hydroxyl, and ether group types, thus suggesting profound degradation processes in the coal.

### Scanning *electron* microscopy

**Figure 9:**
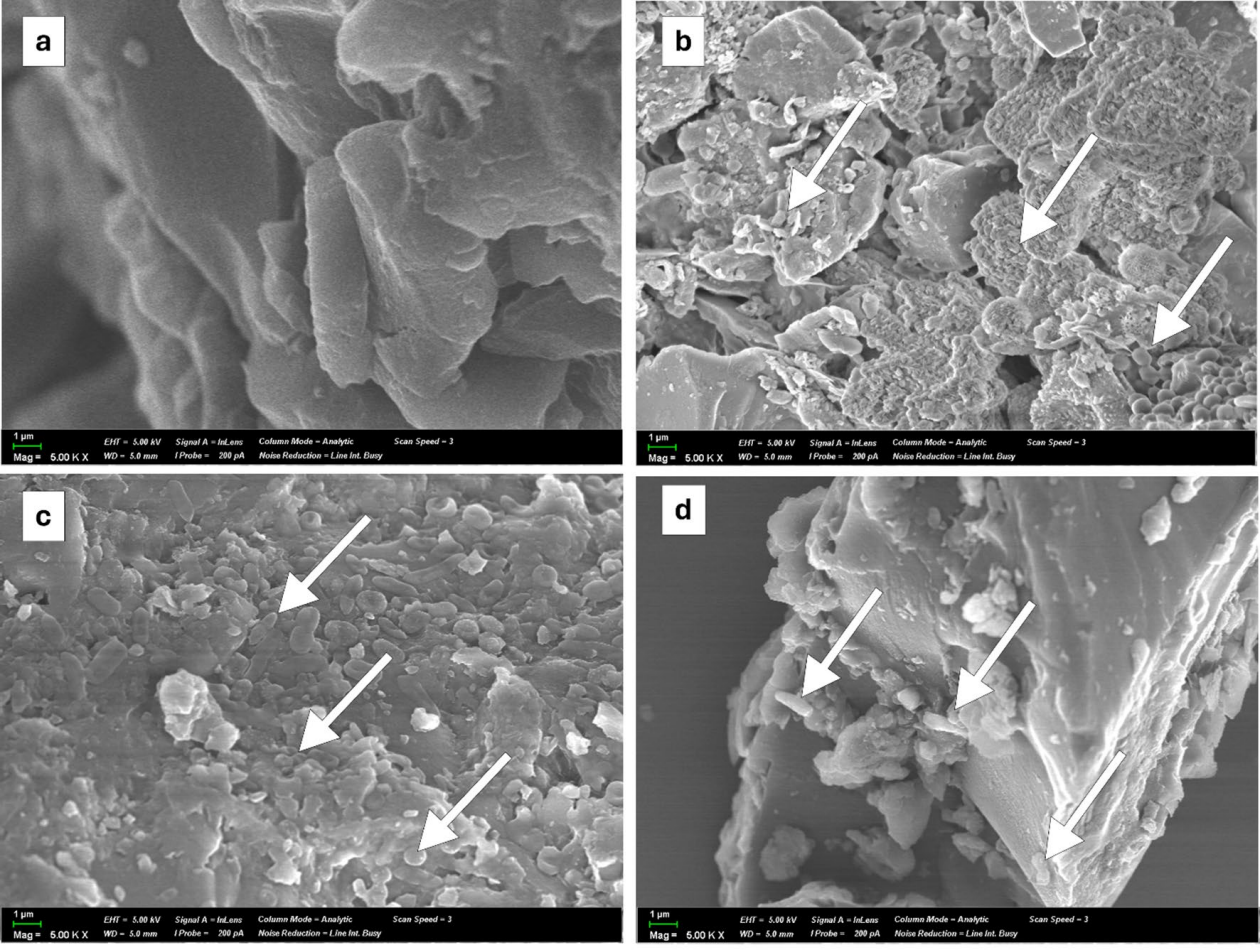
SEM micrographs of raw OIQ (**a**) and bioaugmented coal (**b** EKI, **c** OIQ, **d** KAR) samples.

Figure 9 illustrates the microstructural features of the surfaces of coal samples, both before and after microbial treatment. Prior to treatment, the coal samples exhibited a clean/smooth surface characterized by well-defined boundaries and sharp edges (Fig. 9a). Following coal bioaugmentation, the coal particles displayed a variety of morphologically distinct surface irregularities, including spherical spore-like, coccoid and rod-shaped microbial cells, which resulted in increased roughness of the coal particle surfaces (Fig. 9b–d). Consequently, the edges of the coal particles became smoother. A similar phenomenon was previously observed in a SEM-study and reported by Koca et al.^55^.

## Discussion

Our observations and measurements suggest that coal samples used in this study revealed high biosolubilization and biodegradation potential when treated with activated sludge. Samples susceptibility to microbial attack depends on their elemental (carbon, nitrogen, and oxygen content, as well as the elements ratio) and technical (moisture and organic matter content) characteristics.

The analysis of samples microbial diversity suggests that, at least at the phylum level, native microbial communities of coal and AS are strikingly different. *Actinobacteria*, *Proteobacteria*, and *Acidobacteria* were the main representatives of the microbial community in coal samples and can possess coal degrading capacities^56^. In contrast, the AS was dominated by *Firmicutes* (73.04%).

The comparison between the microbial composition before and after treatment with activated sludge showed that the microbial consortium of AS significantly replaced indigenous microorganisms in the coals. The major phyla such as *Firmicutes*, *Bacteroidetes* were also frequently found in coalbed methane production waters and are known to be involved in coal degradation^56^. Significantly higher quantities of *Firmicutes* and *Bactroidetes* and lower concentrations of *Acidobacteria* and *Chloroflexi* in the AS suggested that activated sludge microorganisms exhibit broader spectrum of metabolic properties and are better adapted to environmental challenges^33^. In particular, the abundance of *Firmicutes* species can strongly facilitate sulfur and iron through redox transformations in coal. These transformations may in turn promote biosolubilization and lead to increased concentrations of soluble α-sulfate or β-iron in LRC^57^. Furthermore, members of *Firmicutes*, especially *Clostridia* are directly involved in the enzymatic break-down of macromolecular compounds and produce acetate, while *Bacteroidetes* as obligate fermenters play central role in metabolism of organic acids and polymers in coal^58,59^.

The CLPP-derived indicators, such as AWCD-, R-, and E-values, are very useful in characterizing the physiological and functional properties of microbial communities. The physiological profile is usually a reliable and convenient predictor of alterations in metabolic activity and/or the possible adaptability of microbial communities under stressful conditions. Numerous studies have demonstrated a tight connection between the fundamental geochemical roles of microorganisms and their metabolic features^60–62^. In our study, because of the high diversity of metabolic pathways involved in the utilization of the substrates used, the microorganisms in coal and AS were found to be primarily heterotrophs. Biochemical relationships and ecological specializations are widely acknowledged as crucial factors for the formation of intricate microbial communities that facilitate the structural and functional degradation of coal^63^.

Rich diversity of physiological properties of AS microorganisms in can greatly enhance the extent and versatility of microbial coal degradation^64^. Therefore, AS can be utilized as a powerful biotechnological tool for effective coal biotransformation within a defined range of parameters^65^. Our results showed that the microorganisms in augmented coal exhibited a much greater biochemical activity than those in raw coals. One of the main markers here was the “high richness index” that indicated many oxidized carbon substrates in bioaugmented coals.

Humic acid-like and fulvic acid-like compounds generated in the coals due to AS-assisted biochemical transformation and representing changes in the macromolecular composition of coal were confirmed by EEM analysis. These findings indicated that the carbon and energy required for microbial growth and the conversion of coal into organic compounds were provided via the selective metabolism of various microorganisms^66^. The IR spectra of raw coals have absorption bands of varying intensities, which ultimately changed after treatment with activated sludge, indicating the positive effect of activated sludge microbial activity to the functional group transformation in the coal. The bands at 1564 and 1421 cm^−1^ found in the coal samples after AS-treatment are rather typical for in high-lignin juvenile coals. This finding is in agreement with the EEM-data and indicates successful “humification” of coal^67^. It was also observed that the signal from hydroxyl stretching vibrations became less prominent for OIQ sample, whereas the intensity of asymmetric =CH_2_ and –CH_3_ bending vibrations and aliphatic =CH– stretching vibrations reduced significantly. These may be indicators for more deeper aerobic oxidation of peripheral non-aromatic structural fragments of coal, leading to higher relative concentrations of carboxyl and carbonyl groups in biomodified coals^68^. We concluded that the highest degree of microbial modifications manifested as IR-spectrum transformation was observed in the OIQ-variant compared to other coal samples. Some unchanged IR-patterns seen in the EKI and KAR variants after bioaugmentation may refer to a more stable nature of these coals.

The adhesion of bacteria to the surface of coal particles and their proliferation as observed directly using SEM brought further direct evidence for successful adaptation of microbial communities to the coal environment, with corresponding utilization of coal components by microorganisms as a metabolic substrate^69^.

## Implications and limitations of this study

The research was unfortunately constrained by the inability to conduct a more comprehensive analysis of the final products of coal biosolubilization and biodegradation, including their quantitative distribution and temporal variations. Nevertheless, the outcomes of this study have the potential to support future efforts focused on the utilization of LRC. We believe that the analysis done with respect to microbial diversity and physiological profile from various coal types and AS may help to get better insights into coal bioprocessing strategies.

## Conclusion

Biodegradation of low-rank coal remains an intriguing scientific problem. The structural and functional degradation of different coal samples carried out using the microbial community of activated sludge has shown first promising results as bioaugmentation method. Our obtained data clearly showed the possibility of stimulating the biodegradation process by exogenous microbial community to reach better coal conversion. Activated sludge showed a high level of physiological and functional activities, which we see as a distinct advantage in selection of efficient coal-processing non-native microorganisms. We think that AS-aided bioaugmentation can develop into a successful eco-friendly biotransformation tool to produce low molecular weight humic compounds for different applications.

## Data Availability

The raw data generated from 16S rRNA gene sequencing and metagenome sequencing have been deposited in the NCBI Sequence Read Archive (SRA) and are available via the BioProject PRJNA1098457.
